# Effect of Anesthetic Technique on the Occurrence of Acute Kidney Injury after Total Knee Arthroplasty

**DOI:** 10.3390/jcm8060778

**Published:** 2019-05-31

**Authors:** Ha-Jung Kim, Hee-Sun Park, Yon-Ji Go, Won Uk Koh, Hyungtae Kim, Jun-Gol Song, Young-Jin Ro

**Affiliations:** Department of Anesthesiology and Pain Medicine, Asan Medical Center, University of Ulsan, College of Medicine, Seoul 05505, Korea; alexakim06@gmail.com (H.-J.K.); lenadiar@naver.com (H.-S.P.); goyunji426@gmail.com (Y.-J.G.); ingwei2475@gmail.com (H.K.); jungol.song@amc.seoul.kr (J.-G.S.); yjro@amc.seoul.kr (Y.-J.R.)

**Keywords:** acute kidney injury, anesthetic technique, total knee arthroplasty

## Abstract

Recent studies have reported the advantages of spinal anesthesia over general anesthesia in orthopedic patients. However, little is known about the relationship between acute kidney injury (AKI) after total knee arthroplasty (TKA) and anesthetic technique. This study aimed to identify the influence of anesthetic technique on AKI in TKA patients. We also evaluated whether the choice of anesthetic technique affected other clinical outcomes. We retrospectively reviewed medical records of patients who underwent TKA between January 2008 and August 2016. Perioperative data were obtained and analyzed. To reduce the influence of potential confounding factors, propensity score (PS) analysis was performed. A total of 2809 patients and 2987 cases of TKA were included in this study. A crude analysis of the total set demonstrated a significantly lower risk of AKI in the spinal anesthesia group. After PS matching, the spinal anesthesia group showed a tendency for reduced AKI, without statistical significance. Furthermore, the spinal anesthesia group showed a lower risk of pulmonary and vascular complications, and shortened hospital stay after PS matching. In TKA patients, spinal anesthesia had a tendency to reduce AKI. Moreover, spinal anesthesia not only reduced vascular and pulmonary complications, but also shortened hospital stay.

## 1. Introduction

Total knee arthroplasty (TKA) is the primary treatment option for advanced inflammatory and degenerative knee osteoarthritis. With an increase in the elderly population, the number of TKAs performed is increasing rapidly, and is expected to continue to increase [1,2]. Even in young patients, the number of TKAs performed is increasing as the surgical technique and durability of the implant have improved [3].

Although controversy remains over the optimal anesthetic technique in perioperative outcomes following TKA, general anesthesia has historically been used for TKA [4]. There is minimal risk of anesthesia failure with general anesthesia; however, although very rare, neuraxial anesthesia, including spinal anesthesia and epidural anesthesia, is associated with neurologic complications, such as radiculopathy, cauda equina syndrome, and paraplegia [5]. In addition, a meta-analysis showed that general anesthesia had no significant risk on perioperative morbidities and mortality compared to regional anesthesia including neuraxial anesthesia and peripheral nerve block in patients undergoing total joint arthroplasty [6]. Moreover, many surgeons and patients were found to prefer general anesthsia over neuraxial anesthesia because of patient anxiety; anesthesiologists also tend to opt for general anesthesia due to more experience with this technique [7]. 

However, there is increasing evidence that neuraxial anesthesia has potential advantages over general anesthesia. Several previous studies reported that neuraxial anesthesia can reduce the risk of deep vein thrombosis, surgical site infection, and perioperative blood loss in orthopedic patients [8,9,10]. A recent study identified that 30-day mortality was also reduced after total joint arthroplasty in patients receiving neuraxial anesthesia [11]. Spinal anesthesia also showed benefits especially in patients with multiple comorbidities [11]. 

Although various studies have analyzed the effect of anesthetic technique on perioperative morbidity and mortality, little is known about the relationship between acute kidney injury (AKI) after TKA and anesthetic technique. The incidence of AKI in patients undergoing TKA is approximately 4–5% and newly developed AKI after noncardiac surgery was found to lengthen postoperative hospital stay and increase mortality [12,13,14]. Thus, identification of modifiable factors associated with AKI is important to improve patient outcomes. 

The current study aimed to identify the influence of anesthetic technique on the incidence of AKI using propensity score (PS) matching. We also evaluated whether the choice of anesthetic technique affected the occurrence of perioperative complications other than AKI.

## 2. Materials and Methods

This study was approved by the Institutional Review Board of Asan Medical Center (2017-0626; date of approval, 29 May 2017) and written informed consent was waived owing to the retrospective nature of this study. All surgeries were performed by three surgeons at a tertiary center in Seoul, Korea.

### 2.1. Study Population

We retrospectively reviewed the medical records of patients who underwent TKA between January 2008 and August 2016. A total of 4702 consecutive TKAs were identified from the electronic medical records system. Of these, 401 TKAs were excluded due to estimated preoperative glomerular filtration rate of less than 60 mL/min/1.73 m^2^, preoperative serum creatinine (sCr) levels greater than 1.5 mg/dL, or a previous history of renal dysfunction. The remaining 4301 TKAs were performed on 2809 patients. In total, 1317 patients underwent a single TKA surgery. The remaining 1492 patients underwent TKAs on both knees; 555 with staggered operations during one hospitalization, 178 with staged operations at intervals of more than two weeks in two separate hospitalizations, and 759 simultaneous operations.

### 2.2. Anesthesia

Standard monitoring was performed in the operating room, including electrocardiogram (ECG), SpO_2_, non-invasive blood pressure, and body temperature. 

In patients receiving TKA under general anesthesia, intravenous propofol (2–3 mL/kg) or thiopental sodium (4–5 mL/kg) with rocuronium (0.6–1.0 mg/kg) was used for induction. After induction, anesthesia was maintained with 1–1.5 minimum alveolar concentration of inhalational agents (sevoflurane or desflurane) with 50% nitrous oxide. Fresh gas flow rate was maintained at 2 L/min and minute ventilation was adjusted with reference to ETCO_2_. Tidal volume was set at 6–8 mL/kg and respiratory rate was set at 10–12/min without positive end-expiratory pressure (PEEP). In patients with decreased SpO_2_, recruitment maneuver was performed and 5 cm H_2_O PEEP was applied. After the procedure was completed, the inhalation agent and nitrous oxide were turned off and the lung was ventilated with 100% oxygen. When an attempt of self-respiration appeared, sugammadex or a cholinesterase inhibitor with anticholinergics was administered and extubation was performed. 

Spinal anesthesia consisted of 10–15 mg bupivacaine and 10–15 mcg of fentanyl intrathecally after a lumbar puncture at the lateral decubitus position. During the intraoperative period, patients were sedated with midazolam and/or dexmedetomidine and breathed 5–6 L/min oxygen via a simple mask. 

In both groups, blood pressure was targeted within normal range. If systolic blood pressure dropped below 80 mmHg, ephedrine (5–10 mg) or phenylephrine (50–100 μg) was injected. In the case of persistent hypotension, continuous infusion of phenylephrine was administered. When the blood pressure was high despite the adequate anesthetic depth and sufficient analgesia, calcium channel blocker or beta blocker was administered considering the underlying disease of the patients and other vital signs upon the decision-making of a staff anesthesiologist. When severe bradycardia (HR < 40 BPM/min) was observed, atropine (0.5 mg) or glycopyrrolate (0.2 mg) was applied. Crystalloid and colloid solution was infused by calculating the maintenance volume and bleeding, and by considering the volume status of the patient. Transfusion was carried out based on the amount of hemorrhage, the symptom of anemia including hypotension, tachycardia, or change of ECG, and the hematocrit level. For postoperative pain control, IV patient-controlled analgesia with fentanyl was available to most patients. 

### 2.3. Clinical Data

We retrospectively obtained demographic, surgical, preoperative, and clinical outcome data of all patients from the electronic medical records system of our institution. The demographic data included age, sex, body mass index (BMI), and the American Society of Anethesiologists’ physical status (ASA PS) classification. Surgical data included the operator and the type of surgical strategy. The surgical strategy was classified as surgery on 1) only one knee (single group), 2) both knees simultaneously (simultaneous group), or 3) both knees sequentially. Patients that underwent surgery on both knees sequentially were divided into two groups according to whether the two surgeries were performed during one or two hospitalization periods (staggered group and staged group, respectively). Preoperative clinical data included medical history, including the presence of diabetes, hypertension, ischemic heart disease, cerebrovascular disease, pulmonary disease, adrenal insufficiency, and the use of the following medications: calcium channel blocker, angiotensin converting enzyme inhibitor/angiotensin receptor blocker, beta blocker, aspirin, antiplatelet agent, HMG-CoA reductase inhibitors, antibiotics, non-steroidal anti-inflammatory drugs, selective COX-2 inhibitor, other analgesics, and steroids. In addition, preoperative laboratory findings, including anemia, platelet, white blood cells, blood sodium level, C-reactive protein (CRP), aspartate aminotransferase (AST), alanine aminotransferase (ALT), albumin, and uric acid, as well as transthoracic/transesophageal echocardiographic findings and pulmonary function test (PFT), were also documented. 

### 2.4. Definitions of Outcomes

The primary outcome of this study was the association of the anesthetic technique with the occurrence of AKI in patients following TKA. AKI was diagnosed based on the Kidney Disease: Improving Global Outcomes (KDIGO) criteria and is described as the change in the sCr level on postoperative days 1 to 7 compared with the baseline sCr level measured before surgery. AKI was defined by alteration of sCr level ≥ 0.3 mg/dL within 48 hours, or a rise in sCr level ≥ 50% within the prior 7 days. 

Intraoperative variables including the use of vasopressor, calcium channel blocker, and beta blocker, the total volume of infused crystalloid and colloid, intraoperative packed red blood cell transfusion, and the lowest mean blood pressure (MBP) were evaluated. In addition, the operation time and the tourniquet time were also calculated. 

Other outcome variables were classified postoperatively as follows: cardiovascular complications, including hypotension, arrhythmia, ischemic heart disease, and congestive heart failure; pulmonary complications, including pneumonia, pleural effusion and pneumothorax; vascular complications, including deep vein thrombosis (DVT) with or without pulmonary thromboembolism (PTE); delirium; cerebrovascular accident; surgical site infection; gastrointestinal complications, including gastric ulcer, diarrhea, and elevated liver enzymes; and urologic complications, including voiding difficulty and urinary tract infection. Major complications, defined as complications requiring a surgical, endoscopic, or radiologic intervention, or those that were life-threatening (Clavien–Dindo classification ≥ 3), were also analyzed. 

### 2.5. Statistical Analysis

The 2809 patients included in this study were classified into four groups based on surgical strategy. Since we aimed to evaluate the risk of AKI during a hospitalization, cases of two TKAs performed on the same patient, staggered and simultaneous, were considered as one case. However, the two TKAs performed on the same patient in the staged group were considered to be two separate cases. Thus, the total number of cases included in the analysis was 2987.

All data are presented as mean ± standard deviation or median (interquartile range) for continuous variables, or number (percentages) for categorical variables. Since the portion of missing data was insignificant, analyses were performed with only the recorded data without any special processing. Demographic characteristics and preoperative laboratory data were compared using Student’s t test or the Mann-Whitney U test for continuous variables. Categorical variables were analyzed with Chi-square or Fisher’s exact test. 

To reduce the influence of potential confounding factors, PS analysis was performed to modify intergroup differences according to the anesthetic technique. Table 1 shows all demographic and perioperative variables used for estimating the PS. Using greedy matching algorithms, we used a caliper of 0.25 standard deviations of the logit of the PS to match patients at a ratio of 1:1. Model discrimination and model calibration were evaluated with c statistics (0.837) and Hosmer-Lemshow statistics (Chi-square = 8.6005, degrees of freedom = 8, *p* = 0.377), respectively. In addition, patients who received both anesthetic techniques were considered self-matching. We evaluated the balance in demographic, surgical, and preoperative covariates of the PS-matched cohort by the standardized mean difference. All absolute standardized differences after PS were less than 0.1. The risk of clinical outcome variables, including occurrence of morbidity, were analyzed by logistic regression using generalized estimating equations in the total and PS-matched cohort. Moreover, hospital stay and postoperative maximum CRP level of each group was compared between the two groups. Statistically significance was set at *p* < 0.05. Statistical analysis was conducted with SAS version 9.4 (SAS Institute Inc., Cary, NC, USA).

## 3. Results

A total of 2809 patients and 2987 cases of TKA were analyzed in this study. Demographic, surgical, and preoperative data of the general anesthesia and spinal anesthesia group without PS matching are shown in Table 1. Without PS matching, the two groups showed significant differences in age, BMI, sex, ASA PS, smoking history, operator, surgical strategy, DM, HTN, IHD, pulmonary disease, clopidogrel, HMG-CoA reductase, NSAIDs, selective COX-2 inhibitor, CRP, AST, albumin, and echocardiographic/PFT findings. After PS matching, standardized differences of all covariates were less than 0.1 (Table 2) and there were no significant differences between the two groups.

Intraoperative data are shown in Table 3. In the analysis of the matched set, the spinal anesthesia group showed significantly fewer requirements of calcium channel blocker and beta blocker, while the lowest MBP was higher. The infused volume of crystalloid and the infused volume of colloid were significantly different, and the volume of urine output also showed a difference between the two groups.

The incidence of AKI, based on the KDIGO criteria, was 162 (5.4%) in all cases (Table 4). Among these, 143 (6.1%) and 19 (3.0%) cases of AKI occurred in the general anesthesia and spinal anesthesia groups, respectively. In a crude analysis of the total set, the spinal anesthesia group demonstrated a significantly lower risk for AKI than the general anesthesia group (Odds ratio (OR) = 0.477, 95% confidence interval [CI] 0.293–0.778, *p* = 0.003). However, in the PS matched set analysis, a total of 37 cases of AKI were included in the PS matched sets and the spinal anesthesia group showed lower incidence of AKI that was not statistically significant (OR = 0.529, 95% CI 0.273–1.024, *p* = 0.059). There were 33 cases of stage 1 AKI (serum creatinine ≥ 1.5–1.9 times baseline or ≥ 0.3 mg/dL increase), out of which 20 cases occurred in the general anesthesia group. The three cases of stage 2 AKI (serum creatinine ≥ 2.0–2.9 times baseline) and one case of stage 3 AKI (serum creatinine ≥ 3.0 times baseline or ≥ 4.0 mg/dL or requirement of renal replacement therapy) occurred only in the general anesthesia group. 

Table 4 shows additional clinical outcomes. Delirium and gastrointestinal complications were significantly more frequent in the spinal versus general anesthesia group in the total set analysis. However, these results were different from the PS-matched set analysis. After PS matching, the general anesthesia group showed a significantly higher risk of pulmonary and vascular complications. Hospital stay was significantly shorter in the spinal anesthesia group compared with the general anesthesia group, in both the total set (spinal anesthesia group 13.6 ± 5.2 days, general anesthesia group 14.0 ± 5.2 days, *p* < 0.001) and the PS matched set (spinal anesthesia group 13.6 ± 5.5 days, general anesthesia group 14.3 ± 5.0 days, *p* = 0.0067). In addition, the patients in the spinal anesthesia group showed significantly lower postoperative maximum CRP levels compared to the general anesthesia group in the total set (spinal anesthesia group 7.1 ± 4.6, general anesthesia group 8.4 ± 5.6, *p* < 0.001) and the PS matched set (spinal anesthesia group 6.9 ± 4.5, general anesthesia group 7.9 ± 5.4, *p* = 0.001).

## 4. Discussion

In the current study, we found that spinal anesthesia might be protective for AKI based on KDIGO criteria in patients undergoing TKA. Moreover, spinal anesthesia had lower observable risks of pulmonary and vascular complications compared to general anesthesia. Hospital stay was also reduced in patients receiving spinal anesthesia. 

Spinal anesthesia showed a trend for a favorable outcome in the occurrence of AKI compared with general anesthesia, although this effect did not reach statistical significance. The protective mechanism of spinal anesthesia is unclear.

First, we suggest that spinal anesthesia may produce these results through sympathetic nerve blockade. The sympathetic nerve fibers originate from the lower thoracic spinal nerves (T7-11). Sympathetic activity is known to have a significant impact on renal function by altering renal hemodynamics, renin release, and sodium handling [15]. Increased sympathetic tone reduces renal blood flow by stimulation of α1 adrenoceptors on renal vasculature and increasing sodium reabsorption by direct effect on the renal tubules [16]. In addition, increased renal sympathetic nerve activity promotes renin release, which is mediated by β-1 adrenoceptors on the juxtaglomerular cells [17]. This series of processes acts to reduce renal blood flow and glomerular filtration rate. In this study, we found that intraoperative urine output was higher in the spinal anesthesia group than in general anesthesia group, even though the amount of fluid administered to both groups was similar. We considered the renal sympathetic nerves blocked, and that the blockade of renal sympathetic activity contributed to the greater urine output, thus resulting in a protective effect for AKI under spinal anesthesia. 

Acute postoperative pain following orthopedic surgery is known to be more severe under general anesthesia compared with spinal anesthesia, with greater opioid use also observed with general anesthesia [18,19,20]. Since the duration of local anesthetics for spinal anesthesia was longer than the duration of TKA, patients generally feel comfortable during the immediate postoperative period. In addition, a previous study suggested that spinal anesthesia lowered the postoperative pain score via preemptive inhibition of afferent nociceptive stimuli and improved organ function [21,22]. Greater postoperative pain in patients receiving general anesthesia increases sympathetic nervous system stimulation, and as a result, the general anesthesia group may have showed a tendency for increased risk for AKI.

A second hypothesis in support of our findings is that the systemic inflammatory response to surgical stress is lower in regional anesthesia than in general anesthesia, resulting in less kidney injury with the spinal technique. The postoperative systemic inflammatory response was reported to be associated with any organ dysfunction, including AKI [23,24]. Inflammatory cytokines inducing neutrophil recruitment and accumulation play a key role in the development of AKI [25,26]. Moreover, the integrity of the endothelial glycocalyx layer, which is closely related to AKI, is disrupted by the inflammatory response [27]. Although controversial, some investigators reported that neuraxial anesthesia suppressed the stress response in elective surgical patients by blocking transmission of pain sensation and sympathetic activation [28,29,30]. In addition, local anesthetics used for neuraxial anesthesia were shown to have intrinsic anti-inflammatory effects [31,32]. In the current study, changes in CRP level revealed that the inflammatory response in the spinal group was reduced compared to the general group, consistent with previous studies. Therefore, we suggest that the reduced inflammatory response to surgical stress by spinal anesthesia might contribute to the trend of decreased risk for AKI. 

Moreover, we found some differences in the intraoperative variables between the two groups. These differences seemed to be derived from the anesthetic techniques since PS matching was performed. A previous study showed that prolonged tourniquet time was related to the increased postoperative complications by leading greater ischemic insult [33]. Therefore, the reduced postoperative complications including AKI in the spinal anesthesia group could be seen as an indirect effect of spinal anesthesia by shortening the tourniquet time. 

Besides the tourniquet time, the amount of fluid also showed differences between the two groups. The infused volume of crystalloid solution was greater in the spinal anesthesia group, while the infused volume of colloid solution was greater in the general anesthesia group. However, the sum of the crystalloid and colloid solution was similar in both groups, so that it was unlikely that the total volume of infused fluid could have had an impact on the occurrence of AKI. In addition, there was also a difference in the lowest MBP between the two groups. Intraoperative hypotension was known to have an association with AKI [34]. However, in this current study, the difference between the two groups was considered clinically insignificant, as the lowest MBP values in each group were higher than 55–60 mmHg, which was considered as cut-off value of hypotensive MBP.

This study showed that spinal anesthesia has advantages over general anesthesia due to reduced postoperative pulmonary complications after TKA. A recent study using a large national database also demonstrated that neuraxial anesthesia decreased the risk of both pulmonary compromise and pneumonia in TKA patients [7]. Respiratory function is disturbed in many aspects from the start of the general anesthesia, and mechanical ventilation under general anesthesia plays a major role in the occurrence of postoperative pulmonary complications [35]. Therefore, we should consider spinal anesthesia first, then general anesthesia, in patients with risk factors for pulmonary complications. 

The beneficial effect of neuraxial anesthesia in vascular complication has been well documented [9,36,37,38,39]. Among them, Memtsoudis et al. suggested that neuraxial anesthesia has a beneficial effect itself beyond simply avoiding general anesthesia, and speculated that this result is due to alteration of the coagulation profile, blood flow, and stress responses to the surgery [7]. However, a number of studies have also shown that anesthetic technique was not associated with the risk of vascular complication, or that neuraxial anesthesia rather increased this risk [8,40,41]. An important factor leading to these inconsistent results was prophylactic treatment with chemical antithrombotics [41]. The studies reporting benefits of neuraxial anesthesia generally included patients that did not receive a prophylactic agent. At our institution, chemical antithrombotic prophylaxis was not applied and our results are consistent with those of previous studies. When chemical antithrombotic prophylaxis is not used, neuraxial anesthesia may be a better choice than general anesthesia. 

Many studies have investigated whether neuraxial anesthesia can reduce the postoperative hospital stay. Although still controversial, a number of studies reported that the length of hospital stay was shorter with neuraxial anesthesia in orthopaedic patients [7,8,42]. Length of hospital stay is a surrogate marker for postoperative complications and use of resources [43]. Thus, the shorter length in the spinal anesthesia group may reflect a better clinical outcome. However, the length of hospital stay was much longer in both the general and spinal groups in this study compared to other studies [7,8,44,45]. This discrepancy is likely due to differences in management protocol. At our institution, patients are discharged after achieving a certain level of rehabilitation. Nonetheless, the spinal anesthesia group did show a shorter length of hospital stay. That is, spinal anesthesia had a beneficial effect on postoperative outcome. 

This study has some limitations. First, there were inevitable flaws due to the nature of the retrospective observational design. We cannot conclude causality between general anesthesia and AKI owing to the characteristics of this study design. Second, there might be other confounding factors that were excluded in the analysis. However, we attempted to include as many factors as possible in the PS matching. Third, our definition of a staged/staggered operation might be different from that of other studies. In the current study, we defined a staggered operation as two operations occurring on two separate days during one hospitalization. However, we focused our investigation on the association of anesthetic technique on AKI, and the surgical strategy was used only for the propensity score matching. Thus, the disparity of the definition of a staged/staggered operation did not likely impact the results. Lastly, since we included patients with normal kidney function in this study, the present findings could not be extrapolated to the patients with decreased renal function. Further studies to evaluate the effect of anesthetic technique on AKI would be required. 

In conclusion, the incidence of AKI, based on the KDIGO criteria, in patients who underwent total knee arthroplasty was 5.4%. Spinal anesthesia had a tendency to reduce AKI. In addition, spinal anesthesia reduced vascular and pulmonary complications compared to general anesthesia. Length of hospital stay was also shortened in patients with spinal anesthesia. 

## Figures and Tables

**Table 1:** Baseline characteristics of all patients included in the total set.

Demographic Data	General (N = 2353)	Spinal (N = 634)	*p*-Value	Standardized Difference
Age (years)	68.6 ± 6.6	69.3 ± 6.4	0.027	0.102
Body mass index (kg/m^2^)	26.8 ± 3.5	26.5 ± 3.2	0.048	0.090
Sex, Female/male	2174 / 179 (92.4/7.6)	568 / 66 (89.6 / 10.4)	0.022	0.092
ASA PS ^†^, 1/2/3	129/2157/67 (5.5/91.7/2.9)	21/581/32 (3.3/91.6/5.1)	0.003	0.152
Smoking History Non/current/ex-smoker	2039/52/262 (86.7/2.2/11.1)	325/19/290 (51.3/3/45.7)	<0.001	0.844
**Surgical Data**				
Surgical strategy, Single/staggered/staged_1^st^/ staged_2^nd^/simultaneous	983/380/149/146/695 (41.8/16.2/6.3/6.2/29.5)	334/175/29/32/64 (52.7/27.6/4.6/5.1/10.1)	<0.001	0.553
Surgeon, B/C/K	719/1173/461 (30.6/49.9/19.6)	326/273/35 (51.4/43.1/5.5)	<0.001	0.542
**Preoperative Medical History**				
Diabetes mellitus	306 (13.0)	105 (16.6)	0.021	0.096
Hypertension	768 (32.6)	261 (41.2)	<0.001	0.173
Ischemic heart disease	164 (7.0)	63 (9.9)	0.012	0.099
Cerebrovascular disease	127 (5.4)	45 (7.1)	0.103	0.066
Pulmonary disease	88 (3.7)	35 (5.5)	0.045	0.078
Adrenal disease	33 (1.4)	7 (1.1)	0.562	0.029
**Preoperative Medication History**				
Calcium channel blocker	927 (39.4)	243 (38.3)	0.625	0.022
Angiotensin-converting enzyme inhibitor	803 (34.1)	220 (34.7)	0.787	0.012
Beta blocker	360 (15.3)	106 (16.7)	0.382	0.038
Aspirin	512 (21.8)	135 (21.3)	0.800	0.011
Clopidogrel	150 (6.4)	58 (9.2)	0.015	0.096
HMG-CoA reductase inhibitors ^‡^	600 (25.5)	234 (36.9)	<0.001	0.236
Antibiotics	12 (0.5)	1 (0.2)	0.323	0.089
Nonsteroidal anti-inflammatory drugs	187 (8.0)	33 (5.2)	0.019	0.123
Selective cyclooxygenase-2 inhibitor	408 (17.3)	140 (22.1)	0.006	0.114
Other analgesics	356 (15.1)	116 (18.3)	0.052	0.082
Steroids	51 (2.2)	19 (3.0)	0.220	0.049
**Preoperative Laboratory Data**				
Anemia	618 (26.3)	147 (23.2)	0.115	0.073
Thrombocytopenia/normal/ thrombocytosis	85/2142/126 (3.6/91.0/5.4)	19/590/25 (3.0/93.1/3.9)	0.255	0.077
Leukopenia/normal/leukocytosis	89/2198/66 (3.8/93.4/2.8)	22/598/14 (3.5/94.3/2.2)	0.657	0.042
Hyponatremia/normal/hypernatremia	26/2279/48 (1.1/96.9/2.0)	10/611/13 (1.6/96.4/2.1)	0.626	0.041
Hemoglobin (g/dL)	12.7 ± 1.1	12.8 ± 1.1	0.111	0.071
C-reactive protein (mg/dL)	0.23 ± 0.48	0.21 ± 0.4	0.014	0.052
Aspartate aminotransferase (IU/L)	23.0 ± 8.9	22.4 ± 9.0	0.008	0.064
Alanine aminotransferse (IU/L)	19.9 ± 11.2	20.4 ± 12.0	0.569	0.039
Albumin (g/dL)	3.9 ± 0.3	3.8 ± 0.3	<0.001	0.418
Uric acid (mg/dL)	4.6 ± 1.2	4.6 ± 1.1	0.519	0.029
Abnormality on echocardiogram	279 (11.9)	48 (7.6)	0.002	0.163
Abnormality on pulmonary function test	103 (4.4)	40 (6.4)	0.041	0.081

Data are presented as mean ± standard deviation or median (interquartile range (IQR)) for continuous variables, or number (percentages) for categorical variables. ^†^ American Society of Anesthesiologists physical status; ^‡^ 3-hydroxy-3-methyl-glutaryl-coenzyme A reductase inhibitor.

**Table 2:** Baseline characteristics of the patients included in the propensity score matched set.

Demographic Data	General (N = 467)	Spinal (N = 467)	Standardized Difference
Age	69.4 ± 6.5	69.3 ± 6.4	0.011
Body mass index (kg/m^2^)	26.4 ± 3.3	26.4 ± 3.3	0.017
Sex, Female/male	423/44 (90.6/9.4)	416/51 (89.1/10.9)	0.049
ASA PS ^†^, 1/2/3	17/425/25 (3.6/91.0/5.4)	18/426/23 (3.9/91.2/4.9)	0.022
Smoking HistoryNon/current/ex-smoker	340/15/112 (72.8/3.2/24.0)	320/18/129 (68.5/3.9/27.6)	0.094
Surgical Data			
Surgical strategy, Single/staggered/staged_1^st^/staged_2^nd^/simultaneous	238/120/19/27/63 (51.0/25.7/4.1/5.8/13.5)	240/110/23/31/63 (51.4/23.6/4.9/6.6/13.5)	0.069
Surgeon, B/C/K	216/208/43 (46.3/44.5/9.2)	204/228/35 (43.7/48.8/7.5)	0.095
Preoperative Medical History			
Diabetes mellitus	74 (15.9)	70 (15.0)	0.023
Hypertension	166 (35.6)	170 (36.4)	0.017
Ischemic heart disease	47 (10.1)	45 (9.6)	0.014
Cerebrovascular disease	29 (6.2)	30 (6.4)	0.008
Pulmonary disease	26 (5.6)	27 (5.8)	0.009
Adrenal disease	7 (1.5)	6 (1.3)	0.020
Preoperative Medication History			
Calcium channel blocker	186 (39.8)	178 (38.1)	0.035
Angiotensin converting enzyme inhibitor	165 (35.3)	162 (34.7)	0.014
Beta blocker	84 (18.0)	77 (16.5)	0.040
Aspirin	101 (21.6)	99 (21.2)	0.010
Clopidogrel	42 (9.0)	39 (8.4)	0.022
HMG-CoA reductase inhibitors ^‡^	160 (34.3)	150 (32.1)	0.044
Antibiotics	0 (0)	1 (0.2)	0.054
NSAIDs	29 (6.2)	24 (5.1)	0.048
Selective cyclooxygenase-2 inhibitor	102 (21.8)	101 (21.6)	0.005
Other analgesics	94 (20.1)	81 (17.3)	0.072
Steroids	13 (2.8)	11 (2.4)	0.025
Preoperative Laboratory Data			
Anemia	119 (25.5)	106 (22.7)	0.066
Thrombocytopenia/normal/thrombocytosis	18/427/22 (3.9/91.4/4.7)	17/431/19 (3.6/92.3/4.1)	0.034
Leukopenia/normal/leukocytosis	25/432/10 (5.4/92.5/2.1)	21/437/9 (4.5/93.6/1.9)	0.043
Hyponatremia/normal/hypernatremia	7/450/10 (1.5/96.4/2.1)	4/455/8 (0.9/97.4/1.7)	0.068
Hemoglobin (g/dL)	12.7 ± 1.2	12.8 ± 1.1	0.088
C-reactive protein (mg/dL)	0.22 ± 0.38	0.23 ± 0.45	0.008
Aspartate aminotransferase (IU/L)	22.2 ± 7.7	22.6 ± 9.6	0.048
Alanine aminotransferse (IU/L)	20.4 ± 12.2	20.5 ± 12.9	0.010
Albumin (g/dL)	3.8 ± 0.3	3.8 ± 0.3	0.085
Uric acid (mg/dL)	4.7 ± 1.2	4.7 ± 1.1	0.003
Abnormality on echocardiogram	49 (10.5)	45 (9.6)	0.032
Abnormality on pulmonary function test	34 (7.3)	34 (7.3)	0.0

Data are presented as mean ± standard deviation or median (interquartile range [IQR]) for continuous variables, or number (percentages) for categorical variables. ^†^ American Society of Anesthesiologists physical status; ^‡^ 3-hydroxy-3-methyl-glutaryl-coenzyme A reductase inhibitor.

**Table 3:** Intraoperative data of the study groups by anesthetic technique in total and matched sets.

Intraoperative Data	Total Set			Matched Set		
	General Group (n = 2353)	Spinal Group (n = 634)	*p*-Value	General Group (n = 467)	Spinal Group (n = 467)	*p*-Value
Use of vasopressor	430 (18.3)	102 (16.1)	0.202	77 (16.5)	86 (18.4)	0.432
Use of calcium channel blocker	366 (15.6)	58 (9.2)	<0.001	65 (13.9)	34 (7.3)	0.001
Use of beta blocker	516 (21.9)	8 (1.3)	<0.001	95 (20.3)	5 (1.1)	<0.001
Red blood cell transfusion	795 (33.8)	103 (16.3)	<0.001	95 (20.3)	99 (21.2)	0.730
Infused crystalloid (mL)	1014.8 ± 600.1	961.1 ± 551.2	0.016	922.4 ± 574.1	1013.5 ± 599.9	0.012
Infused colloid (mL)	544.6 ± 305.1	382.8 ± 209.0	<0.001	496.7 ± 277.9	402.0 ± 215.0	<0.001
Urine output (mL)	193.4 ± 230.8	379.4 ± 350.6	<0.001	168.0 ± 209.9	366.2 ± 341.8	<0.001
The lowest mean blood pressure (mm Hg)	69.3 ± 8.5	73.1 ± 9.8	<0.001	70.3 ± 8.7	72.6 ± 9.9	<0.001
Operation time (minute)	133.4 ± 42.3	122.5 ± 33.8	<0.001	123.7 ± 34.8	124.4 ± 36.9	0.731
Tourniquet time (minute)	123.1 ± 34.5	105.3 ±30.6	<0.001	114.0 ± 31.0	104.5 ± 31.0	<0.001

**Table 4:** Comparison of postoperative clinical outcomes in the study groups by anesthetic technique.

Clinical Outcome	Group	Total Set					Matched Set				
		Event	Odds Ratio	95% Confidence Interval		*p*-Value	Event	Odds Ratio	95% Confidence Interval		*p*-Value
Acute kidney injury	General	143	1				24				
	Spinal	19	0.477	0.293	0.778	0.003	13	0.529	0.273	1.024	0.059
Cardiovascular complication	General	29	1				10				
	Spinal	7	0.895	0.390	2.052	0.793	5	0.495	0.166	1.470	0.205
Pulmonary complication	General	38					14				
	Spinal	7	0.680	0.276	1.676	0.402	4	0.280	0.091	0.862	0.027
Deep vein thrombosis/Pulmonary thromboembolism	General	28					11				
	Spinal	3	0.395	0.119	1.309	0.129	2	0.178	0.039	0.813	0.026
Delirium	General	32					7				
	Spinal	19	2.241	1.254	4.004	0.006	15	2.181	0.873	5.450	0.095
Neurologic complication	General	18					3				
	Spinal	6	1.239	0.490	3.133	0.650	6	2.013	0.497	8.146	0.327
Surgical site infection	General	14					4				
	Spinal	6	1.596	0.611	4.171	0.340	6	1.507	0.420	5.409	0.530
Gastrointestinal complication	General	58					6				
	Spinal	26	1.692	1.060	2.701	0.028	14	2.375	0.943	5.980	0.066
Endocrinologic complication	General	3					2				
	Spinal	1	1.237	0.128	11.918	0.854	1	0.499	0.045	5.540	0.571
Urologic complication	General	24					11				
	Spinal	9	1.397	0.641	3.048	0.400	4	0.358	0.122	1.053	0.062
Major complication	General	70					18				
	Spinal	24	1.283	0.800	2.058	0.301	22	1.233	0.654	2.325	0.517
Intensive care unit admission	General	59	1				15				
	Spinal	21	1.332	0.803	2.210	0.267	19	1.278	0.647	2.526	0.480
